# Supplementary material for: Emerging Role of Oxidative Stress on EGFR and OGG1-BER Cross-Regulation: Implications in Thyroid Physiopathology
Source: Cells. 2022 Feb 26;11(5):822. doi: 10.3390/cells11050822 (PMC8909339; doi:10.3390/cells11050822)
Supplement: Supplementary file 1 [file cells-11-00822-s001.zip › cells-1511929-supplementary.pdf]

Table S1: Details of primers for each evaluated gene by qRT-PCR and gene dosage assay.

Primers for gene expression assay			
Genes	*Forward (5'→ 3')*	*Reverse (5'→ 3')*	*RefSeq mRNA Sequence*
APE1	ACTTCAGGAGCTGCCTGGACT	AATCACCCGGCCTTCCTGATCA	NM_001244249
CXCL8	ATGACTTCCAAGCTGGCCGT	TCCTTGGCAAAACTGCACCT	NM_000584.4
EGFR	CCTGACTCCGTCCAGTCTTG	GCTTGTTACTCGTGCCTTG	NM_005228
ErbB2	TCACCTACAACACAGACACG	GACAGGCAGTCACACAGC	NM_001005862
ErbB3	AGATGCTGAGATAGTGGTGAAG	ATGGTCTTGGTCAATGTCTGC	NM_001982
ErbB4	CAGATGCTACGGACCTTACG	CATTGAAATTGTGCTCCAGTTG	NM_005235
*GUSB**	AGCCAGTTCCTCATCAATGG	GGTAGTGGCTGGTACGGAAA	NM_000181
HO1	CCCAGGCAGAGAATGCTGAGTTC	AGCCTTGCGGTGCAGCTCTTC	NM_002133
JUN/AP1	TCGACATGGAGTCCCAGGA	GGCGATTCTCTCCAGCTTCC	NM_002228
MUTYH	TCGTCTCCCGCCTGAGTCGT	CAGAAGGCTTTGGCCTGACT	NM_001048171
NRF2	TTCAGCCAGCCCAGCACATC	CGTAGCCCGAAGAAACCTCATTGTC	NM_001313902
OGG1	TGGACCTGGTTCTGCCTTCT	TAGCCTGGCTCTTGTCTCCT	NM_016821
PPARG	ACTATGGAGTTCATGCTTGTGA	CCGACAGTACTGACATTTATTTC	NM_001354666
TPO	TCTCATTGGGAAGCAGATGAAG	TCTGTGCATCCGTGAAGACGT	NM_000547
ZEB1	CGCAGTCTGGGTGTAATCGTA	CGTTTCTTGCAGTTTGGGCATT	NM_001128128ì
**Primers for gene dosage assay**			
Genes	*Forward (5'→ 3')*	*Reverse (5'→ 3')*	*RefSeq DNA sequence*
*B-actin**	TACCACTGGCATCGTGATGG	CGGTGAGGATCTTCATCAGG	NC_000007.14
MUTYH			NC_000001.11
Amplicon 1	GAAGCTGCGGGAGCTGAAA	ATCCCCGACTGCCTGAACC	
Amplicon 2	AGCCCCTCTTGGCTTGAGTA	TGCCGATTCCCTCCATTCT	
OGG1			NC_000003.12
Amplicon 1	TCTTTGGGCGTCGACGAGG	GAGGGGACAGGCTTCTCAG	
Amplicon 2	TGTTCAGTGCCGACCTGCGCC	TTTGGAACCCTTTCTGCGCT	

* Housekeeping gene.

Analyzed genes: *APE1*, Apurinic/apyrimidinic Endodeoxyribonuclease 1; *EGFR*, Epidermal Growth Factor Receptor; *ErbB2*, Erb-b2 receptor tyrosine kinase 2; *ErbB3*, Erb-b3 receptor tyrosine kinase 3; *ErbB4*, Erb-b4 receptor tyrosine kinase 4; *GUSB*, Glucuronidase Beta; HO-1), Heme Oxygenase 1; *JUN/AP1*, Jun proto-oncogene, AP-1 transcription factor subunit; *MUTYH*, MutY DNA glycosylase; NRF2, Nuclear Factor, erythroid 2 like 2; *OGG1*, 8-Oxoguanine Glycosylase; *PPARG*, Peroxisome Proliferator Activated Receptor Gamma; *TPO*, Thyroperoxidase; *ZEB1*, Zinc finger E-box Binding homeobox 1.

Figure S1. FACS analysis for FITC-Annexin-V-based apoptosis detection. Untreated Starved NTHY cells. Quiescent NTHY cells treated with H_2O_2 for 15′; Quiescent NTHY cells treated with H_2O_2 for 30′; Untreated Starved TPC1 cells; Quiescent TPC1 cells treated with H_2O_2 for 15′; Quiescent TPC1 cells treated with H_2O_2 for 30′.

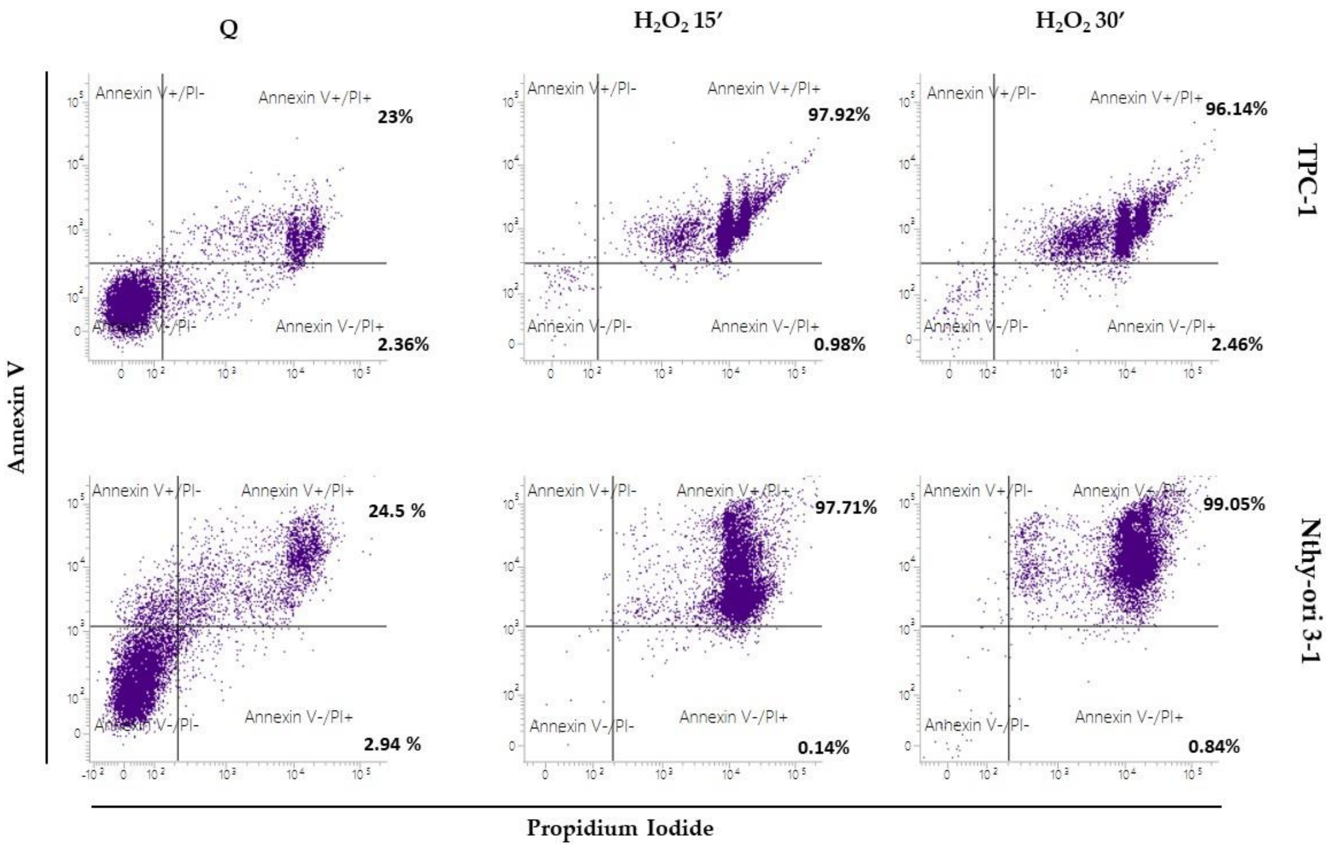

Cell death was assessed on Nthy-ori-3-1 and TPC-1 starvate cells after acute exposure to 10mM H_2O_2 for 15 and 30 minutes. Annexin V (Becton Dickinson Biosciences, San Jose, CA, USA) was used to detect phosphatidylserine presence on cell membranes, in order to discriminate apoptotic cells and counterstained by Propidium Iodide. Each sample (5×10^5 cells) was treated according to the manufacturer's instructions. After staining procedures, samples were analyzed by flow cytometry using a FACSVerse cytometer (Becton Dickinson Biosciences). Finally, data were analyzed using FACSuite v 1.0.6.5230 (Becton Dickinson Biosciences) .. The results were interpreted in the following fashion: cells in the lower-left quadrant (Annexin-V−/PI−) represent living cells; those in the upper-left quadrant (Annexin-V+/PI−) represent early apoptotic cells; those in the upper-right quadrant (Annexin-V+/PI+) represent late apoptotic/death cells. Three independent experiments were performed with three biological replicates per condition each with two technical replicates.

The relative number of apoptotic/death cells as significantly ($p < 0.001$) increased in both cell lines after acute H_2O_2 tratment at 15 and 30 minutes..
